# Health related quality of life in untreated and treated patients with AIS. Study III: trunk appearance and self-image

**DOI:** 10.1186/1748-7161-9-S1-O75

**Published:** 2014-12-04

**Authors:** Manuel Rigo, Monica Villagrasa, Elisabetta DAgata, Beatriz Valer

**Affiliations:** 1 Elena Salva Institut, Barcelona, Spain; 2 Institut Reçerca Vall Hebron, Barcelona, Spain

## Background

The relationship between scoliosis magnitude, HRQL and Self Perception of Trunk Deformity is not clear. To better understand this, a data collection including age, main thoracic and lumbar or thoracolumbar Cob angle, Trunk Asymmetry Perception Scale TAPS, SRS-22 and current treatment was started involving all untreated and treated patients with idiopathic scoliosis attending a rehabilitation clinic, 10 years of age or older at the time of consultation. The data have been retrospectively analyzed and are presented in several studies. This is the study III.

## Purpose

Self- Image in people with Scoliosis is usually the lowest HRQOL factor in comparison to the others. The aim of this study was to assess whether patients with a worse Self-Image might have an “aberrant” perception of their deformity in comparison with people showing a better Self Image.

## Methods

N=240; mean age 19.3 + 10.3 y (10-62), mean Cobb thoracic 33.7º + 13.8, lumbar or thoracolumbar 29.7 + 12.7. SPSS was used for statistics.

## Results

In the group with better Self-Image the correlations between TAPS and Cobb angles (Thoracic: r=-.5; Lumbar/TL: r=-.47) were similar to the ones of the group with worse Self-Image (r=-.5 and r=-.44, respectively).

Furthermore, in the whole population, the correlations between TAPS and Cobb angles (Thoracic: r= -.6; Lumbar/TL: r= -.5) were similar to the correlation between TAPS and SRS-22 SI (r= .5); while the correlations between SI and Cobb Angles (Thoracic: r= -.3; Lumbar/TL: r= -.3) were the lowest ones. All these correlations were statistically significant (p<0.0001).

## Discussion and conclusions

Patients with worse self-Image have an “adequate” perception of the deformity, at least so “adequate” than those with a better self-image, but they have just bigger curves. Moreover the Self Perception of Trunk Deformity measured by TAPS could be considered a variable intermediate between Cobb Angles and Self Image, as TAPS is a recognition of the deformity while SRS-Self Image is a self-evaluation about the body experience, likely influenced by other emotional and cognitive factors.

